# The efficacy and safety of anamorelin for patients with cancer-related anorexia/cachexia syndrome: a systematic review and meta-analysis

**DOI:** 10.1038/s41598-023-42446-x

**Published:** 2023-09-14

**Authors:** Jumpei Taniguchi, Sunao Mikura, Katharina da Silva Lopes

**Affiliations:** Graduate School of Public Health, St. Luke’s International University, Tokyo, Japan

**Keywords:** Cancer therapy, Digestive signs and symptoms

## Abstract

Cancer-related anorexia/cachexia syndrome (CACS) is characterized by anorexia and loss of body weight. Evidence is insufficient to strongly endorse any pharmacologic agent for the treatment of CACS. In this systematic review, we assessed the efficacy of oral anamorelin treatment for patients with CACS. On July 6, 2022, we systematically searched the following databases for randomized controlled trials (RCTs) of adults with CACS comparing oral anamorelin versus placebo: CENTRAL, PubMed, EMBASE, and ICHUSHI. The primary outcomes were total body weight (TBW), patient-reported quality of life (QOL), and adverse events (AEs). Secondary outcomes included lean body mass (LBM), overall survival (OS), non-dominant hand grip strength (HGS), and appetite. We included seven RCTs with a total of 1944 CACS patients. Anamorelin significantly increased TBW (mean difference (MD) 1.73, 95% confidence interval (CI) 1.34–2.13, *p* < 0.00001), LBM (MD 1.06, 95% CI 0.30–1.81, *p* = 0.006), and QOL (standardized mean difference (SMD) 0.16, 95% CI 0.04–0.27, *p* = 0.006) compared with placebo without a significant difference in all AEs, severe AEs, OS, HGS or appetite. Anamorelin may be an effective treatment for CACS patients; however, further studies are needed to confirm the efficacy and safety of this drug.

## Introduction

Cancer-related anorexia/cachexia syndrome (CACS) is a multifocal disease with anorexia and body weight loss associated with reduced adipose tissue and muscle mass^1^. It is frequently seen in patients with advanced cancer, and is estimated to occur in more than 50% of all cancer patients^2^. The criteria for cancer cachexia are loss of body weight of > 5%, or loss of body weight of > 2% in individuals already having depletion based on a current body mass index (BMI) of < 20 kg/m^2^ or skeletal muscle mass^3^. It is related to increased mortality, poor performance status and quality of life (QOL), and poor treatment outcomes, therefore various pharmacological and non-pharmacological treatments are applied^4^. CACS treatment is expected to improve appetite, body weight, QOL, performance status (PS), and overall survival. However, even now there is no effective or standard treatment.

Ghrelin is a 28-amino-acid peptide, and is the natural ligand for the growth hormone secretagogue receptor^5^. Ghrelin is produced in the stomach and stimulates growth hormone secretion and induction of insulin-like growth factor-1 secretion, leading to increased food intake and weight gain^6^. Since previous small randomized trials have shown that ghrelin therapy can be safely administered to patients with advanced cancer, ghrelin-related medicines have recently been studied as a promising approach to CACS^7–9^.

Anamorelin is an oral ghrelin mimetic and selective agonist that exerts its action at the ghrelin receptor^10^. Anamorelin is well tolerated and multiple randomized controlled trials (RCTs) have shown an improvement in total body weight (TBW), lean body mass (LBM), QOL, and appetite in patients with incurable cancer compared with placebo^11–14^. In December 2020, anamorelin 100 mg has been approved for CACS in Japan, largely based on its positive effect on patients’ weight observed in phase II trials; however, anamorelin has not been approved in the United States or Europe, with the Committee for Medicinal Products for Human Use noting its limited effect on lean body mass and no proven impact on hand grip strength or quality of life; additionally, its safety data was inadequately recorded^15^. Therefore, several phase III trials are currently conducted in the United States and Europe.

Two previous systematic reviews from 2017 reported improvement in TBW, LBM, and QOL for cancer patients receiving anamorelin^16,17^. However, limitations such as the lack of a comprehensive search strategy, the small number of eligible studies for inclusion, and heterogeneity of studies have been noted. Several RCTs have also been published since these systematic reviews have been conducted, making it necessary to update the evidence on the effectiveness of anamorelin on TBW, QOL, and adverse events (AEs) in all cancer patients.

The aim of this systematic review is to evaluate the efficacy and limitations of anamorelin on patients with CACS.

## Methods

This systematic review and meta-analysis was carried out according to the PRISMA (Preferred Reporting Items for Systematic Reviews and Meta-Analysis) Statement^18^. The protocol was registered in PROSPERO (CRD42022340705).

### Types of studies

We included individually randomized controlled trials. We excluded animal, in vitro, observational studies, and narrative and systematic reviews.

### Types of participants

We included trials in which the participants were older than 18 years and were patients with any type and stage of cancer.

### Types of interventions and comparison

We compared oral anamorelin (any dosage) with placebo.

### Types of outcome measures

#### Primary outcomes

The primary outcomes were total body weight (TBW), patient-reported quality of life (QOL), and adverse events (AEs). Adverse events were tabulated as all AEs, severe AEs, and drug-related AEs. Adverse events reported as higher than Grade 3 in the Common Terminology Criteria for Adverse Events (CTCAE) score were counted as severe adverse events.

#### Secondary outcomes

Secondary outcomes included lean body mass (LBM), overall survival (OS), non-dominant hand grip strength (HGS), and appetite.

### Search methods for identification of studies

We conducted literature searches for studies in the following electronic databases on July 6, 2022: PubMed, Cochrane Central Register of Controlled Trials (CENTRAL), EMBASE, and ICHUSHI.

The search strategy was based on discussions with an information specialist and was adapted for each database. The full search strategies are detailed in Supplement 1. We only included peer-reviewed articles with human participants, and excluded any abstracts and meeting presentations. No language restrictions were applied.

### Data collection

#### Selection of studies

After database searches and removal of duplicates, JT and MS independently screened and reviewed the title and abstracts of all references identified. The full text of all relevant articles was then retrieved to assess eligibility. Any disagreements were resolved through discussion.

#### Data extraction

JT extracted the following information on each study into a pre-specified data extraction form and SM checked for accuracy: (1) study characteristics: the first author’s name, publication year, type of study, country, and source of funding; (2) patients’ characteristics: cancer type, age, sex, race; (3) intervention and comparison: dose and duration; (4) relevant outcomes.

#### Assessment of risk of bias in included studies

JT and SM independently assessed risk of bias for each study using the revised Cochrane risk of bias tool 2 for randomized trials (RoB 2 tool)^19^. We assessed the following domains: randomization process (D1), deviation from intended interventions (D2), missing outcome data (D3), measurement of outcome (D4), selection of the reported result (D5), and overall bias. Each domain was judged for low or high risk of bias, or any concerns. In accordance with RoB 2, if an individual domain was at a particular level of risk of bias, the overall risk of bias was at least that severe. Therefore, if the risk of bias was “high” in any domain, we judged the overall risk of bias as “high.” If there were “some concerns” in multiple domains, we judged the overall risk of bias as “high” risk for that outcome. We resolved any disagreements through discussion.

#### Assessment of the certainty of evidence

We used the Grading of Recommendations, Assessment, Development, and Evaluations (GRADE) approach^20^ to assess the certainty of the evidence for our outcomes, TBW, QOL, AEs, and LBM using the following five domains: risk of bias, inconsistency, imprecision, indirectness, and publication bias. We judged them to be either of high, moderate, low, or very low certainty. We created a ‘Summary of findings’ table using GRADEpro^21^.

### Meta-analysis and statistical analysis

Statistical analyses and meta-analysis were performed using Review Manager (RevMan) 5.4 software^22^. For continuous outcomes the mean difference (MD) or standardized mean difference (SMD) was calculated with 95% confidence interval (CI). When the standard deviation (SD) was not available, SD was calculated from the standard error (SE) using the RevMan calculator. Hazard ratio (HR) was calculated for expressing OS. Pooled HRs for outcomes were calculated using the inverse variance method. Risk ratio (RR) was used to assess AEs.

We assessed heterogeneity in meta-analysis using Tau^2^, I^2^, and Chi^2^ statistics. If there was no statistical heterogeneity (*p* > 0.1, I^2^ ≤ 50%), we used the fixed-effects model. We used the random-effects model when heterogeneity was detected (*p* < 0.1, I^2^ > 50%).

We performed subgroup analysis according to the dosage of anamorelin (100 mg and 50 mg) to determine whether anamorelin 100 mg, as approved in Japan, is appropriate in terms of efficacy and safety.

## Results

### Study selection

**Figure 1:**
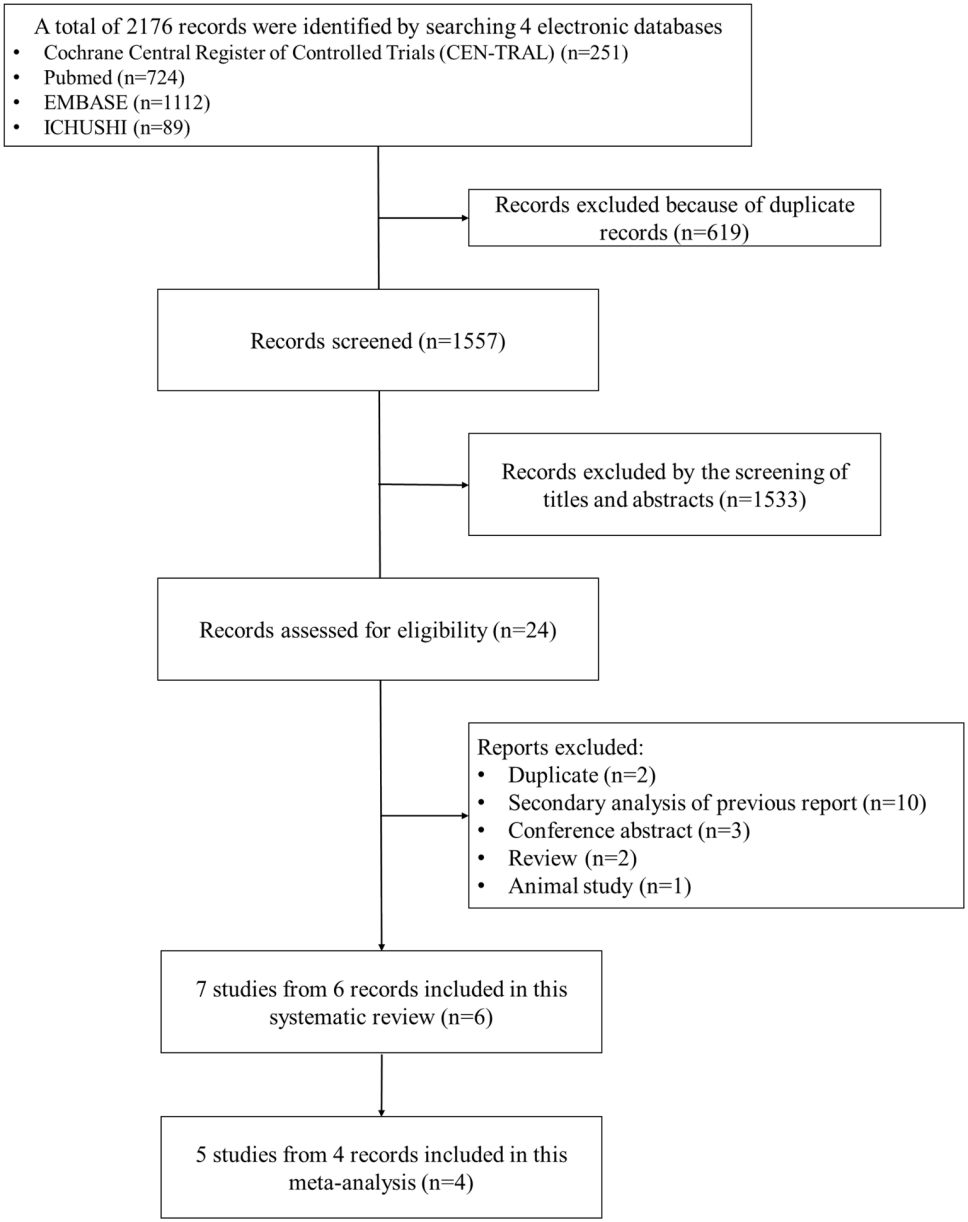
Study flow diagram.

A total of 2176 records were identified by searching four electronic databases. After the exclusion of 619 duplications, we screened 1557 records. We then assessed the full text of 24 articles for inclusion. Ultimately, seven RCTs from six articles were included. The study selection process is shown in Fig. 1.

### Characteristics of included studies

The characteristics of the included studies and patients are presented in Table 1. Among the included RCTs, one RCT was a crossover pilot study^11^, three were phase II clinical trials^12,13,23^, and the remaining three were phase III clinical trials^14,24^. Two RCTs were conducted in the United States^11,12^, two in Japan^13,23^, and the other RCTs were international and multicenter clinical trials conducted in several countries^14,24^. The mean or median age was 60–65 years, with more men in all trials. RCTs conducted in the U.S. or multicounty trials had a higher percentage of white participants, while the RCTs conducted in Japan included only Asians (Japanese). Five RCTs included only NSCLC (non-small cell lung carcinoma) patients^13,14,23,24^ and two RCTs targeted patients with any incurable cancer type^11,12^. The results of two RCTs, Temel 2016 (ROMANA I) and Temel 2016 (ROMANA II), were reported in one article^14^. Currow 2017 (ROMANA III) was a safety extension study of the international phase 3 ROMANA I and ROMANA II trials^24^. The treatment period was 12 weeks in six trials^12–14,23,24^ and only 3 days in the crossover trial^11^. The dose of anamorelin was 50 mg in 3 trials^11–13^ and 100 mg in 5 trials^13,14,23,24^. All RCTs were double-blinded and the intervention was compared to placebo. All outcomes were reported as changes from baseline. Five RCTs were funded by Helsinn Therapeutics (US)^11,12,14,24^and two RCTs by Inc. or Ono Pharmaceutical Co. (Japan), Ltd^13,23^.

**Table 1:** Characteristics of included studies.

Study ID	Study design Phase	Country	Number of participants (male/female)	Age (mean (SD) or median (IQR) age (years)), Race (%)	Cancer type	Intervention and comparison	Available outcomes	Treatment period	Source of funding
Garcia 2013^11^	Randomized crossover study Pilot study	U.S	9 (5/4)	61.9 (mean, ± 10.3), white 7 (77.8%), Black 2 (22.2%), Asian 0	Any incurable cancer	Anamorelin (50 mg) → Placebo	TBW, QOL (ASAS), appetite, AE	3 days	Helsinn Therapeutics (US), Inc
			7 (6/1)	62.9 (mean, ± 8.4), white 4 (57.1%), Black 2 (28.6%), Asian 1 (14.3%)		Placebo → Anamorelin (50 mg)			
Garcia 2015^12^	RCT Phase II	U.S	44 (28/16)	65.5 (median, 19·0–94·0), white 35 (80%), Black 8 (18%), Other 1 (2%)	Any incurable cancer	Anamorelin (50 mg)	TBW, LBM, QOL (ASAS), HGS, AE (all AE, severe AE, drug-related AE), appetite	12 weeks	Helsinn Therapeutics (US), Inc
			38 (23/15)	65.0 (median, 37·0–88·0), white 29 (76%), Black 7 (18%), Other 2 (5%)		Placebo			
Takayama 2016^13^	RCT Phase II	Japan	55 (35/20)	65.7 (mean, ± 8.7), Asian 55 (100%)	NSCLC	Anamorelin (100 mg)	TBW, LBM, QOL (MDASI-J), HGS, AE (all AE, severe AE, drug-related AE), OS, appetite	12 weeks	Ono Pharmaceutical Co., Ltd. Japan
			65 (50/15)	64.8 (mean, ± 8.7), Asian 65 (100%)		Anamorelin (50 mg)			
			60 (39/21)	66.0 (mean, ± 9.4), Asian 60 (100%)		Placebo			
Temel 2016 ROMANA I^14^	RCT Phase III	15 countries	323 (247/76)	63 (median, 30–86), white 319 (99%), Black 1 (< 1%), Asian 1 (< 1%), Other 2 (< 1%)	NSCLC	Anamorelin (100 mg)	TBW, LBM, QOL (A/CS of FAACT), HGS, AE (all AE, severe AE), OS	12 weeks	Helsinn Therapeutics (US), Inc
			161 (121/40)	63 (median, 39–83), White 159 (99%), Black 0 (0%), Asian 0 (0%), Other 2 (1%)		Placebo			
Temel 2016 ROMANA II^14^	RCT Phase III	7 countries	330 (240/90)	63 (median, 36–86), White 326 (99%), Black 2 (< 1%), Asian 0 (0%), Other 2 (< 1%)	NSCLC	Anamorelin (100 mg)	TBW, LBM, QOL (A/CS of FAACT), HGS, AE (all AE, severe AE), OS	12 weeks	Helsinn Therapeutics (US), Inc
			165 (122/43)	62.0 (median, 33–88), white 162 (98%), Black 1 (1%), Asian 1 (1%), Other 1 (1%)		Placebo			
Currow 2017 ROMANA III^24^	RCT Phase III	18 countries	345 (262/83)	62.0 (mean, ± 8.5), no information	NSCLC	Anamorelin (100 mg)	AE (treatment-emergent AEs), TBW, QOL (A/CS of FAACT), HGS	12 weeks	Helsinn Therapeutics (US), Inc
			168 (125/43)	62.2 (mean, ± 8.4), no information		Placebo			
Katakami 2018^23^	RCT Phase II	Japan	84 (59/25)	67.6 (mean, ± 7.9), Asian 84 (100%)	NSCLC	Anamorelin (100 mg)	TBW, LBM, QOL (QOL-ACD), HGS, AE (all AE, severe AE, drug-related AE), appetite	12 weeks	Ono Pharmaceutical Co., Ltd. Japan
			90 (57/33)	67.2 (mean, ± 9.9), Asian 90 (100%)		Placebo			

*A/CS* anorexia/cachexia scale, *AE* adverse event, *ASAS* the Anderson symptom assessment scale, *FAACT* functional assessment of anorexia/cachexia therapy, *HGS* hand grip strength, *LBM* lean body mass, *MDASI* M.D. Anderson Symptom Inventory, *NSCLC* non-small cell lung cancer, *OS* overall survival, *QOL* quality of life, *QOL-ACD* Quality of Life Questionnaire for Patients treated with Anticancer Drugs, *RCT* randomized controlled trial, *TBW* total body weight.

### Risk of bias of included studies

We assessed study quality using RoB 2 tool.

Two trials were judged to be at an overall low risk of bias^14^, three trials were evaluated at a high risk^12,13,23^, and two trials were judged to have some concerns^11,24^. All RCTs employed the random number method according to a computer-generated randomization schedule. Allocation concealment was adequate in all trials. All trials were double-blinded (participants and personnel); however, no RCT reported blinding of the outcome assessor. Three studies had missing outcome data after randomization and were considered at high risk of bias for domain 3^12,13,23^. One study also had moderate outcome data missing, therefore domain 3 was judged to be of some concern^24^. One study was judged as high risk of bias for domain 5 since it was unclear whether the data was analyzed in accordance with a pre-specified analysis plan and there were multiple eligible outcome measurements within the outcome domain^23^. Similarly, two studies did not indicate a data analysis plan, therefore we judged domain 5 to have some concerns^11,13^. The risk of bias assessment for each RCT is summarized in Table 2.

**Table 2:**
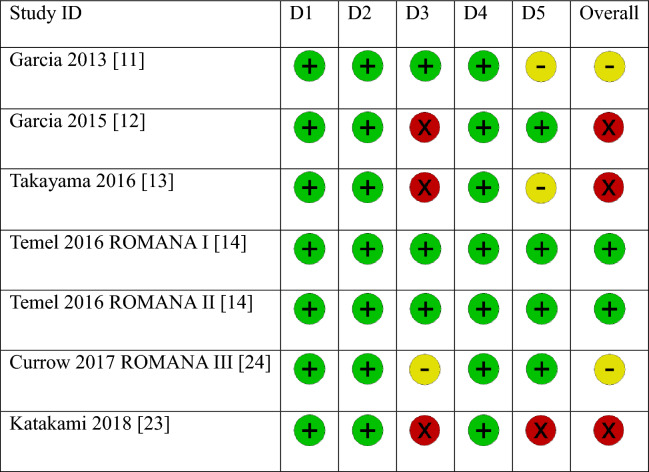
Risk of bias summary.

D1: Bias arising from the randomization process.

D2: Bias due to deviation from intended interventions.

D3: Bias due to missing outcome data.

D4: Bias in measurement of the outcome.

D5: Bias in selection of the reported result.

### Effects of intervention

We summarized the certainty of the evidence for TBW, LBM, QOL, and AEs using the GRADE approach, as shown in Table 3. We downgraded by one or two levels because of high risk of bias due to missing outcome data and reporting bias or the small number of studies and wide CIs. The certainty of the evidence was moderate for TBW, all AEs, severe AEs, and low for LBM, QOL, and drug-related AEs.

**Table 3:**
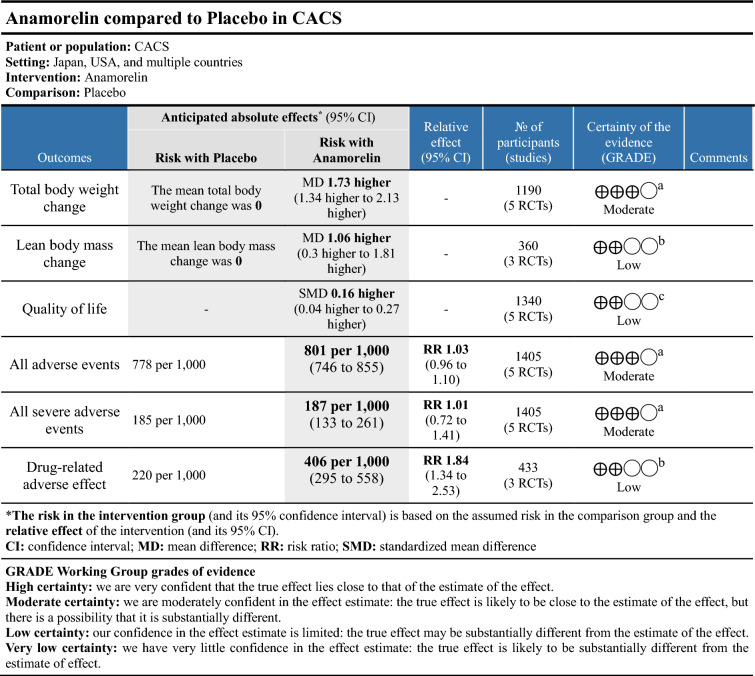
Summary of findings.

^a^Downgraded by one level because the included RCTs were of high risk of bias due to missing outcome data.

^b^Downgraded by two level because the included RCTs were of high risk of bias due to missing outcome data, and small sample size with wide CI.

^c^Downgraded by two level because the included RCTs were of high risk of bias due to missing outcome data and reporting bias.

### Primary outcomes

#### Total body weight

**Figure 2:**
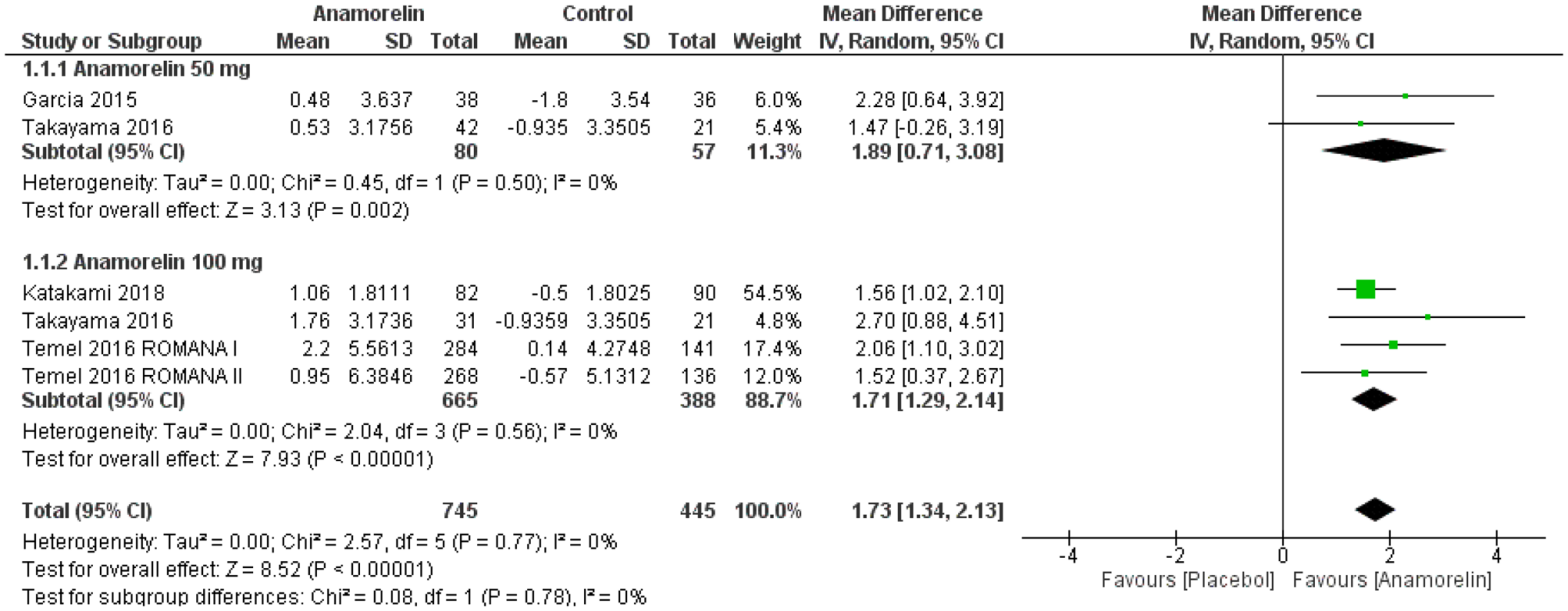
Forest plots showing the effect of anamorelin on total body weight compared with placebo.

Five RCTs with 1190 patients were included in the analysis of TBW change from the baseline (Fig. 2)^12–14,23^. There was a significant increase in TBW from baseline among participants treated with anamorelin (MD 1.73, 95% CI 1.34–2.13, *p* < 0.00001; moderate-certainty evidence). Subgroup analysis according to dosage revealed no differences between 50 and 100 mg of anamorelin. We did not detect heterogeneity (Chi^2^ = 2.57, *p* = 0.77, I^2^ = 0%). Garcia 2013 reported TBW from baseline at 3 days; therefore, the study was not included in the meta-analysis^11^. The study reported a statistically significant average weight gain of 1.0 kg in the anamorelin group compared with the placebo control (95% CI 0.30–1.9, *p* = 0.016). Currow 2017 was a safety 12 weeks extension study of Temel 2016 ROMANA I and ROMANA II; therefore, we did not include this trial in this meta-analysis^24^. Over the 0–24 weeks treatment period, TBW significantly increased in the anamorelin group versus the placebo group (MD 2.1 kg, 95% CI 1.3–3.0, *p* < 0.001).

#### Quality of life

**Figure 3:**
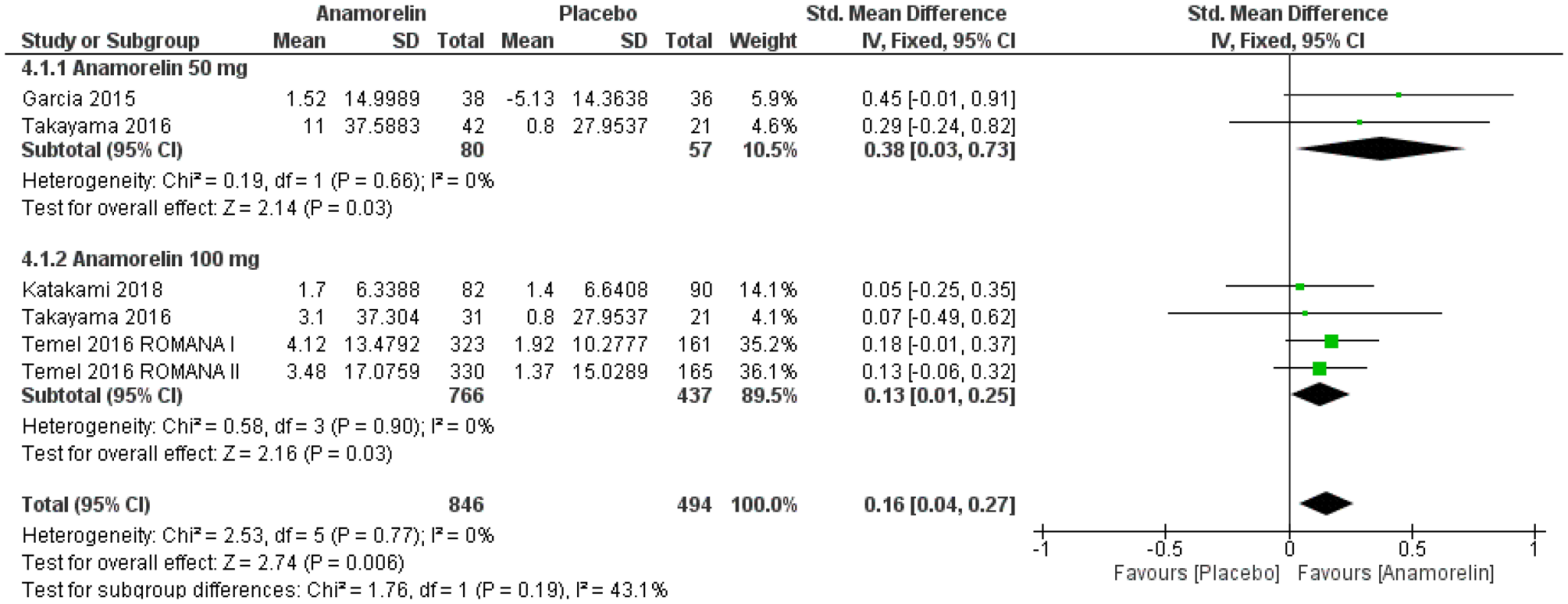
Forest plots showing the effect of anamorelin on quality of life compared with placebo.

Five studies with a total of 1340 participants reported QOL (Fig. 3)^12–14,23^. The results showed anamorelin significantly improved QOL compared with placebo (SMD 0.16, 95% CI 0.04–0.27, *p* = 0.006; low-certainty evidence). There were no differences between subgroups. Quality of life was assessed with the adjusted Anderson symptom assessment scale (ASAS) in one RCT^12^, the M.D. Anderson Symptom Inventory (MDASI) scale in one RCT^13^, the anorexia/cachexia scale of functional assessment of anorexia/cachexia therapy (A/CS of FAACT) in two RCTs^14^, and quality of life questionnaire for patients treated with anticancer drugs (QOL-ACD) scale in one RCT^23^. Heterogeneity was low (Chi^2^ = 2.53, *p* = 0.77, I^2^ = 0%). Garcia 2013 reported the mean-adjusted ASAS total scores from baseline at 3 days; therefore, we did not include this study in the meta-analysis^11^. The results of this study showed the mean-adjusted ASAS total scores significantly increased from 66.44 to 73.80 for the intervention group compared with 66.44–67.44 for the placebo group (95% CI 3.5–10.3, *p* < 0.002). Currow 2017 reported QOL as the Anorexia/Cachexia Subscale score^24^. However, there were no significant differences between the two groups (MD 1.2, 95% CI − 0.1 to 2.5) over the period of 24 weeks.

#### Adverse events and drug-related adverse events

**Figure 4:**
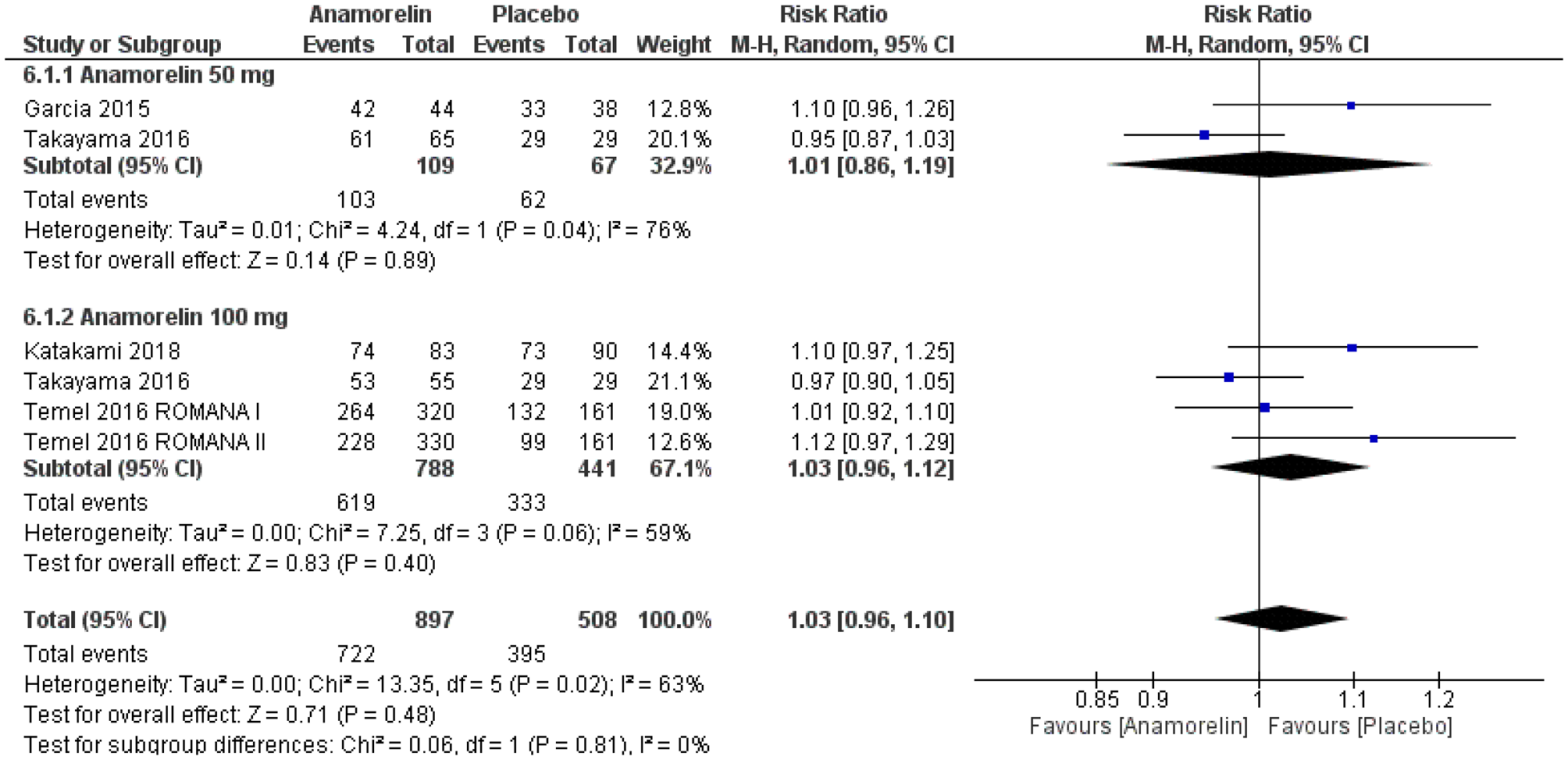
Forest plots showing the effect of anamorelin on all adverse events compared with placebo.

**Figure 5:**
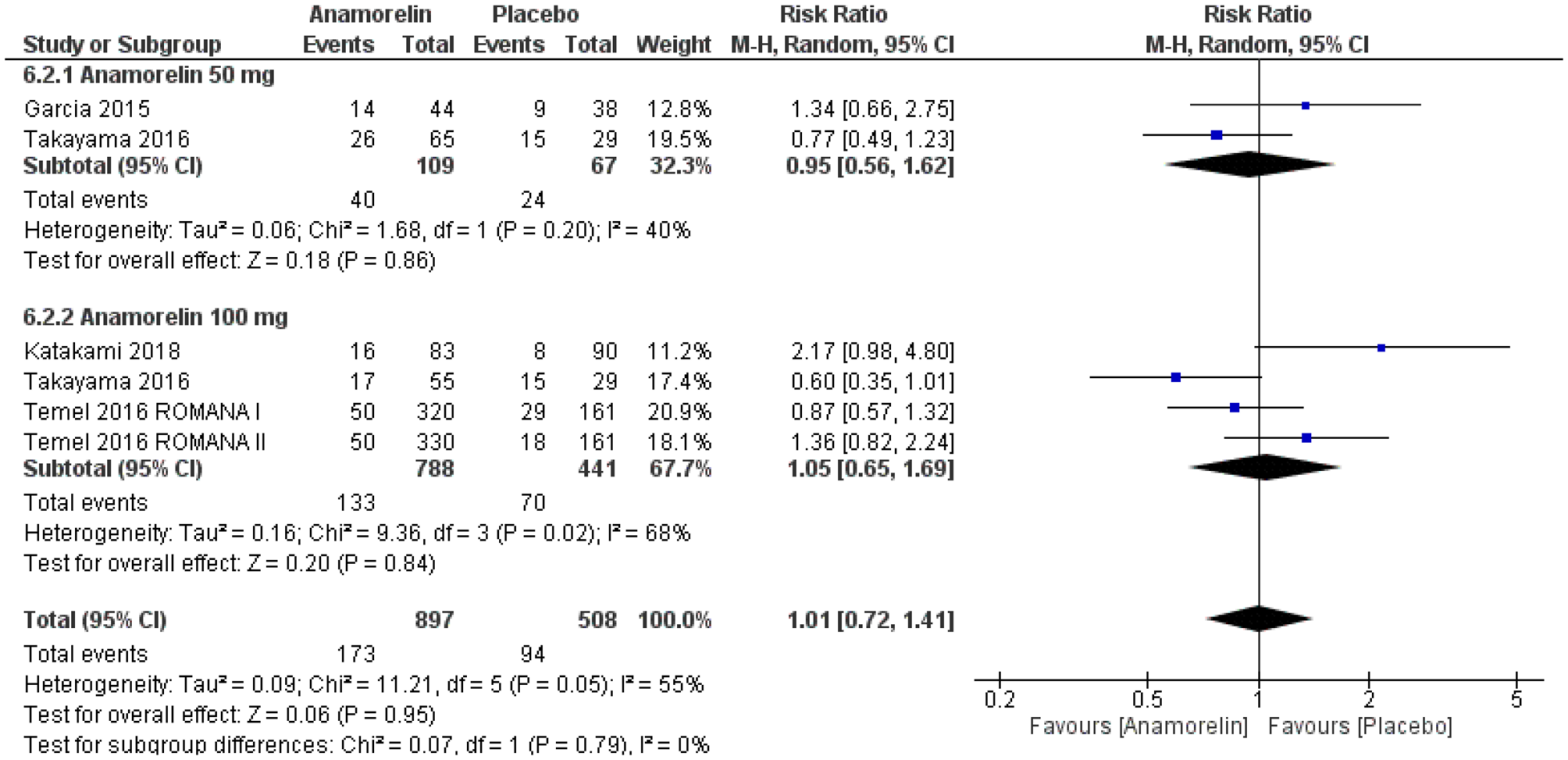
Forest plots showing the effect of anamorelin on severe adverse events compared with placebo.

A total of 1405 patients from five RCTs were included in this analysis (Figs. 4 and 5)^12–14,23^. The frequency of all AEs did not differ significantly between the anamorelin and placebo group (RR 1.03, 95% CI 0.96–1.10, *p* = 0.48; moderate-certainty evidence). The frequency of severe AEs also did not differ significantly between the two groups (RR 1.01, 95% CI 0.72–1.41, *p* = 0.95; moderate-certainty evidence). We detected high heterogeneity for all AEs and severe AEs (Chi^2^ = 13.35, *p* = 0.02, I^2^ = 63%; Chi^2^ = 11.21, *p* = 0.05, I^2^ = 55%, respectively).

**Figure 6:**
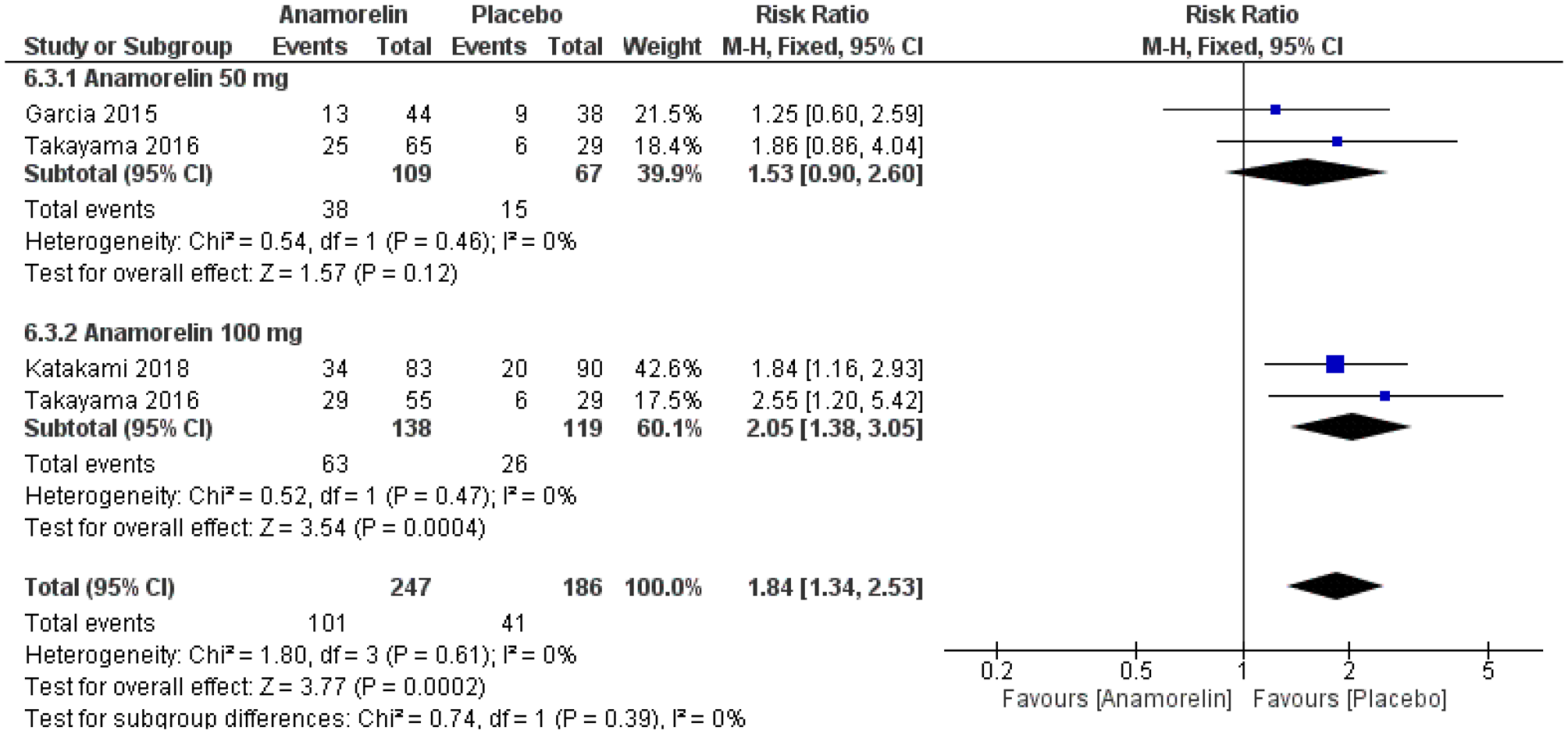
Forest plots showing the effect of anamorelin on drug-related adverse events compared with placebo.

A total of 433 patients with 142 drug-related adverse events were reported in three RCTs (Fig. 6)^12,13,23^. The frequency of drug-related adverse events was significantly higher in the anamorelin group compared with the placebo group (RR 1.84, 95% CI 1.34–2.53, *p* = 0.0002; low-certainty evidence). There was no heterogeneity (Chi^2^ = 1.80, *p* = 0.61, I^2^ = 0%). Garcia 2013 was not included in this meta-analysis because the total duration of anamorelin and placebo treatment was 6 days^11^. Twelve of the 16 patients (75%) reported more than one AE while receiving anamorelin and eight (50%) while receiving placebo. There were no serious AEs while receiving anamorelin, and there was one severe AE while receiving placebo. Adverse events in four patients were assessed to be possibly or probably related to anamorelin. Currow 2017 was a safety extension study of the international phase 3 Temel trials) and therefore, we did not include this study in the meta-analysis^24^. Adverse events were reported as treatment-emergent AEs (TEAEs). In this study, 179 of 343 patients (52.2%) in the anamorelin group and 93 of 167 patients (55.7%) in the placebo group reported any TEAEs, with 44 (12.8%) and 21 (12.6%) serious TEAEs. Of these, 12 (3.5%) and 2 (1.2%) were determined to be drug-related AEs, respectively. Overall, authors concluded that there are no major safety concerns.

### Secondary outcomes

#### Lean body mass

**Figure 7:**
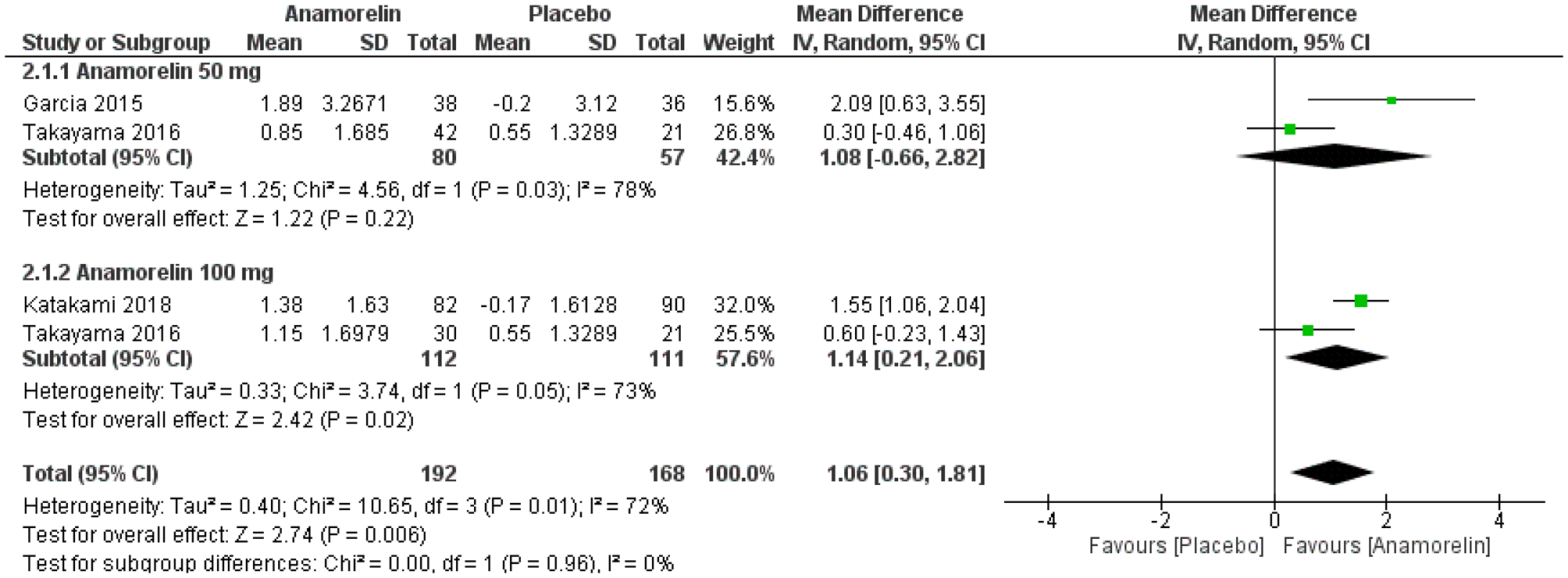
Forest plots showing the effect of anamorelin on lean body mass compared with placebo.

We included three RCTs with 360 patients in this meta-analysis of LBM (Fig. 7)^12,13,23^. There was a significant increase in LBM for patients receiving anamorelin compared with placebo (MD 1.06, 95% CI 0.30–1.81, *p* = 0.006; low-certainty evidence). We detected high heterogeneity between included studies (Chi^2^ = 10.65, *p* = 0.01, I^2^ = 72%). ROMANA I and ROMANA II reported LBM as median values and were not included in this meta-analysis^14^. However, anamorelin revealed a significant increase in LBM over 12 weeks in both ROMANA I (anamorelin group 0.99 kg (median), 95% CI 0.61–1.36 vs placebo group -0.47 kg (median), 95% CI − 1.00 to 0.21) and ROMANA II (anamorelin group 0.65 kg (median), 95% CI 0.38–0.91 vs placebo group -0.98 kg (median), 95% CI − 1.49 to − 0.41).

#### Non-dominant hand grip strength

**Figure 8:**
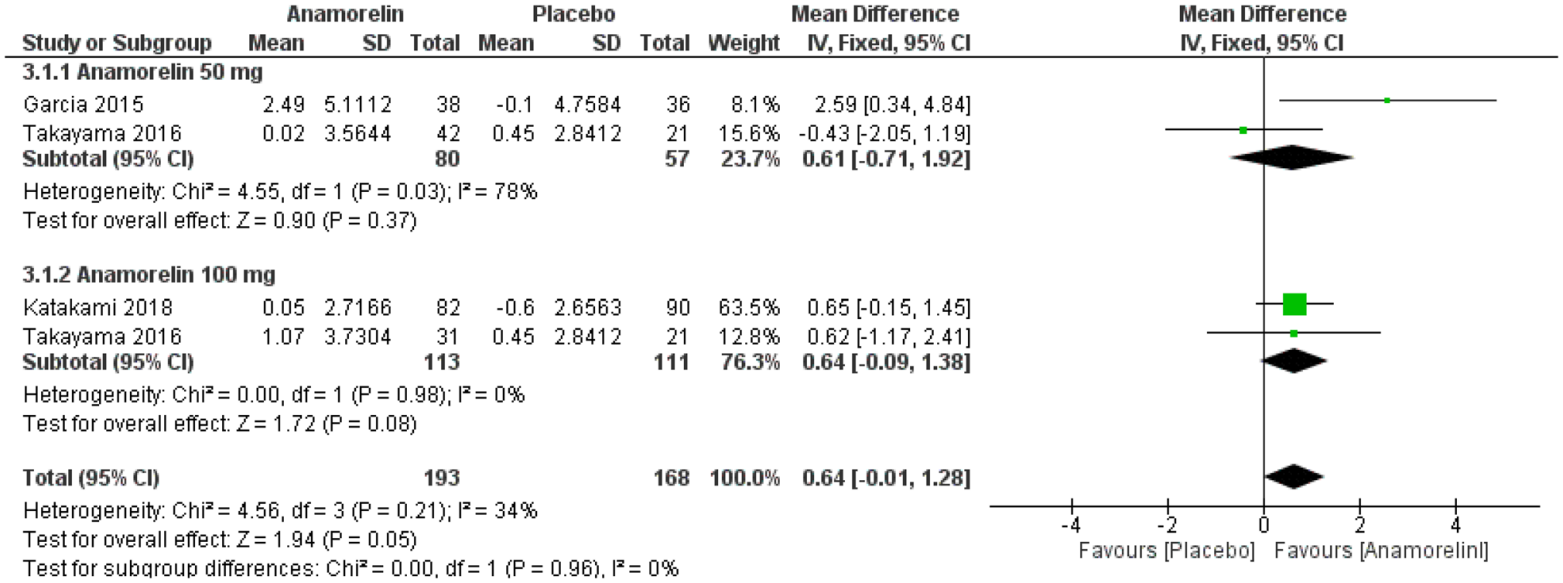
Forest plots showing the effect of anamorelin on non-dominant hand grip strength compared with placebo.

We included three RCTs with 361 patients for HGS in this meta-analysis (Fig. 8)^12,13,23^. There were no significant differences between the anamorelin group and the placebo group (MD 0.64, 95% CI − 0.01 to 1.28, *p* = 0.05), and there were no differences between subgroups. Heterogeneity was moderate (Chi^2^ = 4.56, *p* = 0.21, I^2^ = 34%). ROMANA I and ROMANA II reported HGS as median values and therefore, were not included in this meta-analysis^14^. Anamorelin 100 mg did not show any benefits in ROMANA I (anamorelin group − 1.10 kg, 95% CI; − 1.69 to − 0.40 vs placebo group − 1.58 kg, 95% CI − 2.99 to − 1.14) and ROMANA II (anamorelin group − 1.49 kg, 95% CI − 2.06 to − 0.58 vs placebo group − 0.95 kg, 95% CI − 1.56 to 0.04). The extension study of ROMANA I and ROMANA II also did not show any benefits at 24 weeks^24^.

#### Overall survival

**Figure 9:**
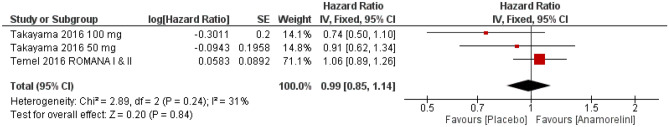
Forest plots showing the effect of anamorelin on overall survival compared with placebo.

We included two RCTs in this analysis (Fig. 9)^13,14^. Both of these two RCTs evaluated OS at 1 year after 12 weeks of anamorelin treatment. The pooled HR was not significantly different between the intervention group and the placebo group (HR 0.99, 95% CI 0.85–1.14, *p* = 0.84). Heterogeneity was moderate between included studies (Chi^2^ = 2.89, *p* = 0.24, I^2^ = 31%).

#### Appetite

**Figure 10:**
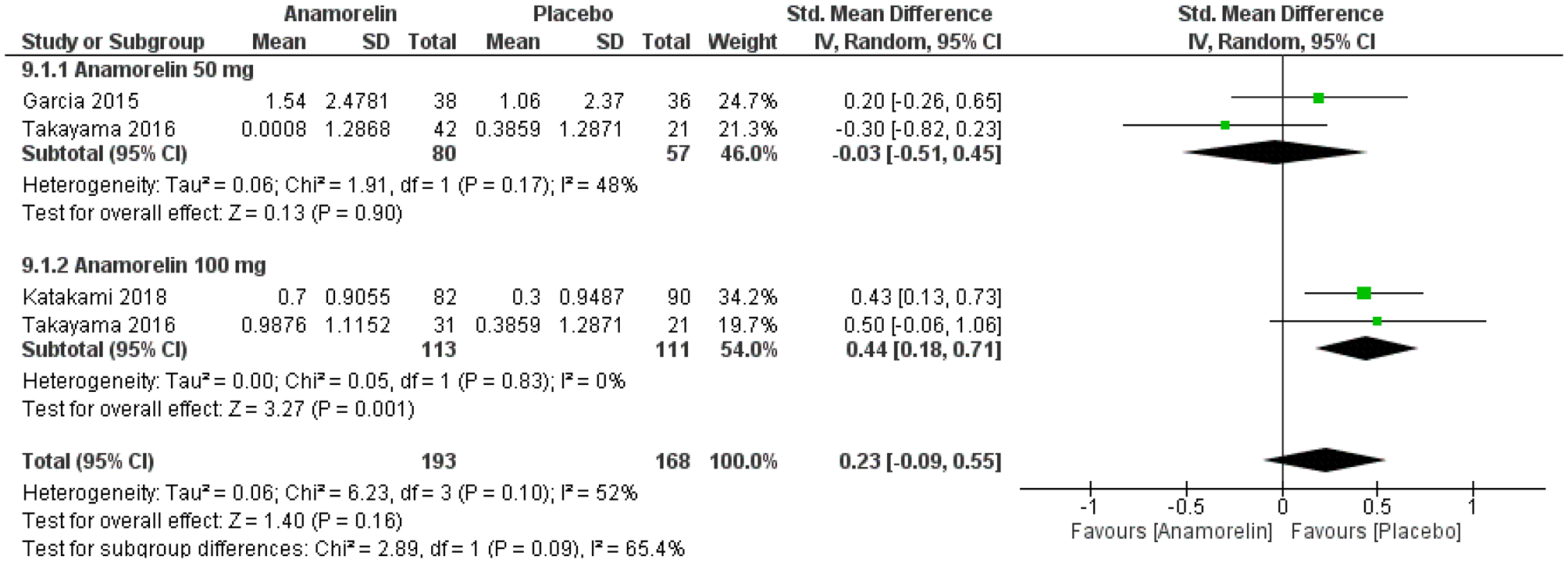
Forest plots showing the effect of anamorelin on appetite compared with placebo.

Data on appetite were available for 361 individuals in three RCTs (Fig. 10)^12,13,23^. Patients receiving 100 mg of anamorelin showed a significant improvement in appetite compared with control (SMD, 0.44, 95% CI 0.18–0.71, *p* = 0.001). However, there were no differences between the groups when patients received 50 mg of anamorelin (SMD, − 0.09, 95% CI − 0.44 to 0.26, *p* = 0.62). There was no significant difference between subgroups according to the dose of anamorelin. Overall, we detected high heterogeneity between studies (Chi^2^ = 6.23, *p* = 0.10, I^2^ = 52%).

## Discussion

We conducted a systematic review and meta-analysis of RCTs to determine the clinical benefit of anamorelin for CACS patients. Our results suggest that anamorelin improves TBW, LBM, and QOL without increasing systemic AEs from soon after the start to 12 weeks of treatment. There were no significant differences between subgroups (50 mg and 100 mg) for all outcomes. Anamorelin significantly increased drug-related adverse events; however, there were no significant dose-dependent differences between subgroups. No significant improvement in OS or HGS was identified; however, results are derived from a small number of trials.

We assessed the certainty of evidence for the outcomes TBW, AEs, LBM, and QOL using GRADE (Table 3). None of the included studies had concerns about random allocation, blinding, or outcome measures. However, some studies were of high risk of bias due to missing outcome data and reporting bias. We also had a limited number of studies for the outcomes LBM and drug-related AEs, so the small number of participants may have resulted in imprecise effect estimates, as indicated by wide CIs. Therefore, we downgraded by one or two levels. Overall, the certainty of the evidence was moderate for TBW, all AEs, severe AEs, and low for LBM, QOL and drug-related AEs.

A previous systematic review from 2017 compared anamorelin versus placebo and included four RCTs^17^. The review showed an increase in LBM (SMD 0.34, 95% CI 0.21–0.46, *p* < 0.00001) and TBW (SMD 1.91, 95% CI 0.53–3.29, *p* = 0.007) from baseline, and improved the total ASAS score (MD 8.05, 95% CI 5.97–10.12, *p* < 0.00001). However, high heterogeneity was observed because of the inclusion of Garcia 2013 in the meta-analysis, a trial where patients received anamorelin for only 3 days. Notably, the review also focused on the molecular mechanisms, presenting increases in IGF-1, which has anabolic and appetite-stimulating effects, and IGFBP-3, which indicates an increase in protein synthesis. The positive effects of anamorelin on TBW, LBM might be mediated through these molecular mechanisms. Since all four RCTs in the review were included in our analysis, the results of the review were similar to our results. Our review further reported about QOL, OS, and drug-related AEs by adding three additional RCTs.

Another systematic review conducted in the same year also compared anamorelin to placebo^16^. This review included six RCTs; four RCTs from peer-reviewed articles and two reports from annual meetings. Anamorelin did not cause an increase in AEs compared with placebo, significantly increased TBW (MD 1.78, 95% CI 1.28–2.28, *p* < 0.00001) and LBM (MD 1.10, 95% CI 0.35–1.85, *p* = 0.004), and improved QOL (SMD 0.19, 95%CI 0.08–0.30, *p* = 0.0006). No significant improvement in OS was observed. The authors suggested that anamorelin 100 mg was the appropriate dose mainly based on the subgroup analysis of appetite that 100 mg significantly improved appetite compared to 50 mg. The results of this review were generally consistent with our results, because all four RCTs from peer-reviewed articles were included in our analysis, and two reports from annual meetings were presumed to be prior reports of the RCTs included in our analysis. However, our study did not show significant differences between subgroups in all outcomes, including appetite. Therefore, it is still unclear whether 100 mg of anamorelin is an appropriate dose. In comparison to this systematic review, we included an additional trial and the two reports from annual meetings which have now been published in peer-reviewed journals. Moreover, we assessed the certainty of the evidence using the GRADE approach^20^.

There is still no established, high-quality, evidence-based drug treatment for patients with CACS. Loss of body weight in CACS is a marker of both progression of the syndrome and prognosis, and there has also been a trend towards lower response rates when using chemotherapy among loss of body weight patients with CACS^1^. Therefore, various drug treatments have been studied in the hope of increasing appetite and weight gain. Two relatively well-studied options are available: progesterone analogs and glucocorticoids^25^. Progesterone analogs improve appetite and body weight in patients with CACS, but the type of weight gain is primarily due to gain in adipose tissue rather than skeletal muscle^26^. They have also been associated with serious AEs such as thromboembolic events, edema, adrenal suppression, and increased mortality, suggesting that their administration must balance potential benefits and risks^27^. Glucocorticoids have been suggested to improve appetite to a similar degree to that seen with progesterone analogs^28^. However, it has not been shown to increase body weight, and given a wide variety of other side effects such as myopathy, infections, and hyperglycemia, and decline in efficacy associated with long-term use, the role of glucocorticoids as an appetite stimulant is sometimes limited in patients with a life expectancy of a few weeks to several months^25^. Moderate to low evidence indicates that, anamorelin increased TBW, LBM, and QOL without increasing overall AEs over a 12-week treatment period. The mechanism of action may be related to a dose-dependent increase in growth hormone, IGF-1, and IGFBP-3 concentrations after treatment with anamorelin as shown in previous research in healthy volunteers^13^.

Our results have important clinical implications for healthcare providers. Anamorelin may be a valuable treatment option for patients suffering from CACS.

### Limitations

We thoroughly assessed the current literature to study the efficacy of anamorelin for CACS. However, our study has several limitations. First, the number of studies for inclusion and available data was limited. We identified seven RCTs from six articles, one of which was a pilot study completed within 3 days of the observation period^11^, and one of which was an extension of two RCTs that were reintegrated^24^. Therefore, we could not include them in this meta-analysis. ROMANA I and ROMANA II, which were considered to have the lowest risk of bias and highest reliability, reported results of LBM and HGS in a way that could not be used for meta-analysis^14^. Regarding OS, which is the most important outcome, only two RCTs reported results at 12 months after treatment^13,14^. Therefore, long-term benefits of treating CACS with anamorelin is still unclear.

Second, although this study demonstrated significant improvements in TBW and QOL, it is not yet clear whether these changes were clinically meaningful. For example, patients had increased TBW with anamorelin, however, caloric intake or food diaries were not reported. This may limit our ability to estimate the effect of anamorelin on food intake. Although each study reported patients’ QOL, comparison was limited because studies used different tools to assess QOL. Furthermore, several studies measured multiple QOL outcomes and gave priority to reporting those that showed significant improvement^13,14,23^.

Third, due to the characteristic of CACS, there are some missing data that may be due to death or changes in physical condition, causing selection bias. In addition, included studies focused mainly on NSCLC. The efficacy of anamorelin for other types of cancer is still unclear, therefore limiting the external validity of the intervention.

Despite these limitations, we were able to show that anamorelin improved TBW, LBM, and QOL without increasing AEs in CACS patients.

### Implications for practice

In this systematic review and meta-analysis of RCTs on anamorelin, we showed that anamorelin improves TBW, LBM, and QOL in patients with CACS without increasing AEs. Although anamorelin 100 mg is currently approved, there is room for further study regarding the appropriate dose, as subgroup analyses of all outcomes showed no significant differences.

We showed that anamorelin would be a valuable option for CACS, for which few effective treatments are available. The evidence is still limited, but several international clinical trials are currently in progress (NCT04844970, NCT03637816, NCT03743064, NCT03743051), which will provide us with more robust evidence in the future. In addition to weight gain and QOL, future studies should ideally also assess the appropriate dose, chemotherapy response, and long-term OS. Since we were only able to demonstrate safety until 12 weeks in this analysis, it is necessary to conduct studies to confirm long-term safety. Since many RCTs only included NSCLC patients, it would also be necessary to confirm whether similar results can be obtained in populations with other types of cancer.

## Conclusions

Moderate to low evidence suggests that anamorelin increases TBW, LBM, and QOL in patients with CACS without increasing overall AEs. Anamorelin may be one of the effective options for CACS patients; however, further studies are needed to confirm the efficacy and safety of this drug.

### Supplementary Information

Supplementary Information.

## Data Availability

Data used and analyzed was extracted from primary studies included in this review. The data analyzed is available from the review authors on request.
