# Supplementary material for: The efficacy and safety of anamorelin for patients with cancer-related anorexia/cachexia syndrome: a systematic review and meta-analysis
Source: Sci Rep. 2023 Sep 14;13:15257. doi: 10.1038/s41598-023-42446-x (PMC10502008; doi:10.1038/s41598-023-42446-x)
Supplement: Supplementary file 1 — Supplementary Information. [file 41598_2023_42446_MOESM1_ESM.docx]

**Supplementary Information**

**Database search strategies**

**CENTRAL**

1. ((cancer* OR tumor* OR “Tumor” OR “Neoplasm*” OR “malignan*” OR “Anorexia*” OR “Cachexia” OR “Carcinoma” OR “Appetite loss” OR “weight loss”)) :ti, ab, kw

2. Mesh descriptor: [Neoplasms] explode all trees

3. Mesh descriptor: [Anorexia] explode all trees

4. Mesh descriptor: [Cachexia] explode all trees

5. (#1 or #2 or #3 or #4)

6. ((“Anamorelin” OR “Adlumiz”)):ti, ab, kw

7. Mesh descriptor: [Ghrelin] explode all trees

8. Mesh descriptor: [Receptors, Ghrelin] explode all trees

9. (#6 or #7 or #8)

10. (#5 and #9)

**Pubmed**

1. ("cancer*"[Title/Abstract] OR "tumor*"[Title/Abstract] OR "Tumour"[Title/Abstract] OR "Neoplasm*"[Title/Abstract] OR "Neoplasms"[MeSH Terms] OR "malignan*"[Title/Abstract] OR "Anorexia"[MeSH Terms] OR "Anorexia*"[Title/Abstract] OR "Cachexia"[Title/Abstract] OR "Cachexia"[MeSH Terms] OR "Carcinoma"[Title/Abstract] OR "Appetite loss"[Title/Abstract] OR "Weight loss"[Text])

2. ("Anamorelin"[Title/Abstract] OR "Ghrelin"[MeSH Terms] OR "Ghrelin"[Title/Abstract] OR "Adlumiz"[Title/Abstract] OR "Anamorelin"[Supplementary Concept] OR "receptors, ghrelin"[MeSH Terms])

3. ("randomized controlled trial"[Publication Type] OR "controlled clinical trial"[Publication Type] OR "Randomized"[Title/Abstract] OR "Placebo"[Title/Abstract] OR "drug therapy"[MeSH Terms] OR "Randomly"[Title/Abstract] OR "Trial"[Title/Abstract] OR "Groups"[Title/Abstract]) NOT ("animals"[MeSH Terms] NOT "humans"[MeSH Terms])

4.(#1 and #2 and #3)

**Embase**

(((cancer:ab,ti OR tumor*:ab,ti OR tumour:ab,ti OR neoplasm:ab,ti OR malignan*:ab,ti OR anorexia*:ab,ti OR cachexia:ab,ti OR 'appetite loss':ab,ti OR 'weight loss':ab,ti) OR ('neoplasm'/exp OR 'anorexia'/exp OR 'cachexia'/exp OR 'body weight loss'/exp)) AND ((anamorelin:ab,ti OR ghrelin:ab,ti OR adlumiz:ab,ti) OR ('ghrelin'/exp OR 'ghrelin receptor'/exp OR 'anamorelin'/exp))) AND (('randomized controlled trial'/de OR 'controlled clinical study'/de OR random*:ti,ab OR 'randomization'/de OR 'intermethod comparison'/de OR placebo:ti,ab OR compare:ti OR compared:ti OR comparison:ti OR ((evaluated:ab OR evaluate:ab OR evaluating:ab OR assessed:ab OR assess:ab) AND (compare:ab OR compared:ab OR comparing:ab OR comparison:ab)) OR ((open NEXT/1 label):ti,ab) OR (((double OR single OR doubly OR singly) NEXT/1 (blind OR blinded OR blindly)):ti,ab) OR 'double blind procedure'/de OR 'parallel group*':ti,ab OR crossover:ti,ab OR 'cross over':ti,ab OR (((assign* OR match OR matched OR allocation) NEAR/5 (alternate OR group* OR intervention* OR patient* OR subject* OR participant*)):ti,ab) OR assigned:ti,ab OR allocated:ti,ab OR ((controlled NEAR/7 (study OR design OR trial)):ti,ab) OR volunteer:ti,ab OR volunteers:ti,ab OR 'human experiment'/de OR trial:ti) NOT ((random* NEXT/1 sampl* NEAR/7 ('cross section*' OR questionnaire* OR survey* OR database*)) NOT ('comparative study'/de OR 'controlled study'/de OR 'randomi?ed controlled':ti,ab OR 'randomly assigned':ti,ab) OR ('cross-sectional study'/de NOT ('randomized controlled trial'/de OR 'controlled clinical study'/de OR 'controlled study'/de OR 'randomi?ed controlled':ti,ab OR 'control group*1':ti,ab)) OR (((case NEXT/1 control*):ti,ab) AND random*:ti,ab NOT 'randomi?ed controlled':ti,ab) OR ('systematic review':ti NOT (trial:ti OR study:ti)) OR (nonrandom*:ti,ab NOT random*:ti,ab) OR 'random field*':ti,ab OR (('random cluster' NEAR/3 sampl*):ti,ab) OR (review:ab AND review:it NOT trial:ti) OR ('we searched':ab AND (review:ti OR review:it)) OR 'update review':ab OR ((databases NEAR/4 searched):ab) OR ((rat:ti OR rats:ti OR mouse:ti OR mice:ti OR swine:ti OR porcine:ti OR murine:ti OR sheep:ti OR lambs:ti OR pigs:ti OR piglets:ti OR rabbit:ti OR rabbits:ti OR cat:ti OR cats:ti OR dog:ti OR dogs:ti OR cattle:ti OR bovine:ti OR monkey:ti OR monkeys:ti OR trout:ti OR marmoset*:ti) AND 'animal experiment'/de) OR ('animal experiment'/de NOT ('human experiment'/de OR 'human'/de))))

**ICHUSHI**

(((((腫瘍/TH or 腫瘍/TA) or (癌/TA) or ((悪液質/TH or 悪液質/AL)) or ((食欲不振/TH or 食欲不振/AL)) or (食思低下/AL) or ((体重減少/TH or 体重減少/AL))) and (((Anamorelin/TH or アナモレリン/AL)) or ((Anamorelin/TH or anamorelin/AL)) or ((Anamorelin/TH or エドルミズ/AL)) or ((Anamorelin/TH or Adlumiz/AL)) or ((Ghrelin/TH or グレリン/AL)) or (("Ghrelin Receptors"/TH or グレリン受容体/AL))))) and (PT=原著論文))
